# Facial phantom model: a low-cost and safe tool for teaching botulinum toxin application in neurology residencies

**DOI:** 10.1055/s-0044-1779037

**Published:** 2024-01-29

**Authors:** Rodrigo Alencar e Silva, Pedro Fraiman, Clécio de Oliveira Godeiro Júnior

**Affiliations:** 1 Universidade Federal do Rio Grande do Norte, Hospital Universitário Onofre Lopes, Departamento de Neurologia, Natal RN, Brazil.; 2 Universidade Federal de São Paulo, Departamento de Neurologia e Neurocirurgia, São Paulo SP, Brazil.

**Keywords:** Botulinum Toxins, Hemifacial Spasm, Blepharospasm, Simulation Exercise, Motor Skills, Toxinas Botulínicas, Espasmo Hemifacial, Blefaroespasmo, Exercício de Simulação, Destreza Motora

## Abstract

**Background** The application of botulinum toxin (BoNT) in the treatment of blepharospasm and hemifacial spasm (HS) is a well-established practice. However, neurology residency programs often rely on real patients for training, which has limitations in terms of patient availability and skill acquisition.

**Objective** Assess the efficacy of a new facial phantom model for acquiring motor skills in BoNT application.

**Methods** An anthropomorphic facial phantom model was developed in collaboration with a medical training simulator start-up. A group of seven neurologists and one ophthalmologist with expertise in BoNT application evaluated the model using an adapted learning object review instrument (LORI). The instrument assessed aspects such as: content quality, alignment of learning objectives, feedback and adaptation, motivation, presentation design, and accessibility.

**Results** The facial phantom model received high scores in the LORI evaluation, with the highest ratings given to alignment with learning objectives and motivation. The model also scored well in terms of accessibility, content quality, and presentation design. However, feedback and adaptation received a lower score due to the static nature of the model.

**Conclusion** The facial phantom model shows promise as a valuable tool for teaching and developing competence in BoNT application for HS and blepharospasm. The model reduces the reliance on real patients for training, providing a broader and safer learning experience for neurology residents. It also provides a realistic learning experience and offers portability, cost-effectiveness, and ease of manufacturing for use in various medical training scenarios. It is an effective and accessible tool for teaching BoNT application.

## Resumo

**Antecedentes** A aplicação de toxina botulínica (TxB) no tratamento do blefaroespasmo e do espasmo hemifacial (EH) é uma prática bem estabelecida. No entanto, os programas de residência em neurologia frequentemente dependem de pacientes reais para treinamento, o que apresenta limitações em termos de disponibilidade de pacientes e aquisição de habilidades.

**Objetivo** Avaliar a eficácia de um novo modelo de manequim facial para aquisição de habilidades motoras na aplicação de TxB.

**Métodos** Foi desenvolvido um modelo antropomórfico de manequim facial em coloboração com uma empresa de simuladores de treinamento médico. Um grupo constituído por sete neurologistas e um oftalmologista com experiência em aplicação de TxB avaliou o modelo utilizando um instrumento adaptado de revisão de objeto de aprendizagem (LORI). O instrumento analisou aspectos como: qualidade do conteúdo, alinhamento dos objetivos de aprendizagem, feedback e adaptação, motivação, concepção da apresentação e acessibilidade.

**Resultados** O modelo de manequim facial obteve pontuações altas na avaliação do LORI com os maiores escores em alinhamento com os objetivos de aprendizagem e motivação. O modelo também obteve boas pontuações em termos de acessibilidade, qualidade do conteúdo e concepção da apresentação. No entanto, o item *feedback* e adaptação recebeu uma pontuação média mais baixa, devido à natureza estática.

**Conclusão** O modelo manequim facial mostra-se promissor como uma EH e blefaroespasmo. O modelo reduz a dependência de pacientes reais para treinamento portátil, de baixo custo e de fácil fabricação para uso em diversos cenários de treinamento, proporcionando uma experiência de aprendizagem mais ampla e segura para residentes de neurologia. Além disso, fornece uma experiência de aprendizagem realista e oferece portabilidade, economia e facilidade de fabricação para uso em vários cenários de treinamento médico. É uma ferramenta eficaz e acessível para o ensino da aplicação de TxB.

## INTRODUCTION

The intramuscular application of botulinum toxin (BoNT) is the treatment of choice for blepharospasm (BS) and hemifacial spasm (HS). 1 The long-term efficacy and safety of BoNT have been evaluated and widely accepted in the treatment of these diseases. 1 2 3

In Brazilian neurology residency programs where there is a specialized clinic for training in BoNT application, the acquisition of manual skills for BoNT application is typically performed on the sick patient. The most common initial form of technique learning is through the study of human anatomy atlases and the visualization of the procedure performed by the mentor. The acquisition of motor skills is obtained directly from the patient under supervision and feedback from the preceptor. Thus, training will depend on the presence of patients with the diseases, their willingness to undergo treatment by professionals improving their technique, and the number of times the procedure is performed. These variables can be excluded with simulation-based teaching-learning.

A survey of neurology and child neurology residents who were members of the American Academy of Neurology found that 66% of them reported a desire for additional training in the technique of BoNT application. Regarding their experience with BoNT application, 39.2% had performed the procedure more than once, 16.4% had performed it only once, 33.3% had only observed the procedure, and 11.1% had no access to any form of training. 4

It is unquestionable that repetition and training activity are important factors in promoting long-term knowledge retention, both for low and high-complexity skills. Regarding learning curves in simulation-based education and motor skills training, there is assumed to be a dose-response relationship, with an increasing number of repetitions resulting in a greater performance improvement until a plateau is reached. 5

Simulation-based education is a tool capable of providing effective and active acquisition of skills and competencies, reducing unfavorable outcomes in the real world. In addition, simulation is a form of learning with situations close to reality, which relates to retention of knowledge for a longer period and absorption of content in a more pleasant and enjoyable way than usual education. The importance of this research regarding practical training in neurology residencies and postgraduate programs in Brazil is to provide, through the development of a facial simulator, efficient and more comfortable learning for the physician and patient in acquiring the technique of BoNT application in HFS and BSP.

## METHODS

This study was an interventional study designed to assess a new anthropomorphic facial phantom model for acquiring motor skills in the technique of chemical block with BoNT in HS and BS.

### Anthropomorphic simulator of the facial region

**Figure 1:**
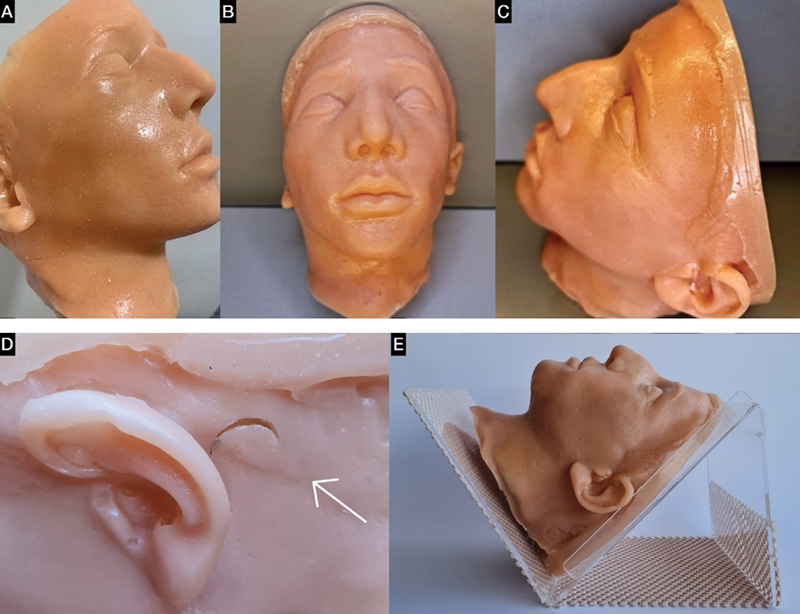
Anthropomorphic facial and cervical simulator. ( **A, B, C** ) Customized mannequin with external anatomical markers for facial characterization. ( **D** ) Monoblock model with elastomer skin, gel elastomer subcutaneous tissue, and polyurethane bone structure layers. Retroauricular drainage points. ( **E** ) Acrylic base with non-slip rubber fabric for stable positioning in supine or sitting positions.

The model was developed by the authors in collaboration with a local low-cost medical training realistic phantom simulator start-up (Medskills, Natal, Brazil). An anthropomorphic simulator of the facial region and part of the antero-superior cervical region was created by customizing a commercial mannequin ( Figure 1A ). The process of artistic modeling was used to characterize a young adult with external anatomical markers such as eyebrows, ears, closed eyes, and facial expression at rest, as is commonly performed during the procedure. The anatomical boundaries of the phantom model included the implantation of hair superiorly, retroauricular regions laterally, and the anterosuperior portion of the cervical region inferiorly. ( Figure 1B and 1C )

A silicone and polyurethane mold was produced that involved the surface of this area of the mannequin. From the mold that was created, the monoblock model was produced in three layers (elastomer, gel elastomer, and polyurethane), and the colors of the layers were obtained from the use of pigments for human skin and subcutaneous tissue. After the mold removal procedure, using a standardized cutting instrument, drainage openings were made in the retroauricular regions

The simulator weighs 742g, with a height and width of 21cm and 16cm, respectively. The lower part is 16cm in the anteroposterior direction and 11cm wide. It is equipped with an outer layer that mimics the skin, made of elastomer, an intermediate layer made of gel elastomer, which represents the subcutaneous tissue, and a third, more internal layer, which corresponds to the bone structure, made of polyurethane. Between the intermediate and internal layers, there is a virtual space that can serve as a reservoir for the liquid injected into the gel elastomer during training. The drainage points for draining the applied liquid are located in the retroauricular regions ( Figure 1D ).

In addition to this model, there is an acrylic base surrounded by a non-slip rubber fabric to reproduce the supine or sitting position for performing the procedure ( Figure 1E ).

### Simulation of botulinum toxin injection techniques

The model was tested and peer-evaluated by a group of seven neurologists and one ophthalmologist who had at least six months of practice in the application of BoNT. Each participant completed one hands-on session.

In addition to the facial simulator and acrylic support, participants were given a pair of gloves, sterilized insulin syringes and needles, and ampoules of saline solution. The evaluators were instructed to simulate BoNT applications commonly performed in the pathologies under study.

Subsequent to the BoNT application procedure, the evaluation instrument for the learning object attached - LORI - was provided to participants to evaluate the characteristics of the learning object, which was to be filled out.

### Adapted LORI assessment

The participants evaluated the model using an adapted learning object review instrument (LORI) ( Supplementary Material - https://www.arquivosdeneuropsiquiatria.org/wp-content/uploads/2023/10/ANP-2023.0163-Supplementary-Material.docx ). 6 This study surveyed the responses of the evaluators, and the adapted LORI contained eight questions about six aspects: content quality, alignment of learning objectives, feedback and adaptation, motivation, presentation design, and accessibility. These aspects were scored on a five-level value scale for each item. In this evaluation instrument, items 6 and 8, which refer to ease of use and interactive compliance with standards, respectively, were considered not relevant, and therefore, the option “not applicable” (NA) was chosen for these items.

In each item, the characteristics of the learning objective (LO) were evaluated on a five-level value scale, from 1 to 5, and the higher the score, the more relevant the LO was considered. The item analyzed on Content Quality refers to the balanced presentation of ideas, accuracy, appropriate levels of detail, and the possibility of reuse in different contexts. The other topic that relates to the Alignment of LOs concerns whether the object is aligned with the LOs, that is, in this context, if it is appropriate for physicians to develop the technical skill of applying BoNT in HE and SB. Feedback and adaptation are applied, in this analysis, in relation to the LO being able to provide instruction, simulate or build phenomena in response to the developed manual skill. Another aspect analyzed was in relation to Motivation, which is up to the LO to have the ability to motivate and interest a group and, in addition, to have the possibility of offering learning based on real life. Presentation Design is about producing visual information for enhanced learning and effective mental processing. Finally, Accessibility deals with the possibility of the LO presentation format providing accommodation to different professionals, whether they have a physical disability or mobility difficulties.

The company which provided the phantom simulator did not participate in the evaluation. The authors do not have shares and did not receive any compensation from the company.

### Standard protocol approvals, registrations, and patient consents

IRB approval (CAEE: 57574022.8.0000.5292) was obtained within Universidade Federal do Rio Grande do Norte's ethical standards committee. Voluntary participation was considered consent. This study did not include patients or animals. We used SQUIRE checklist 7 when writing our report.

### Data availability

Anonymized data not published within this article will be made available by request from any qualified investigator.

## RESULTS

**Figure 2:**
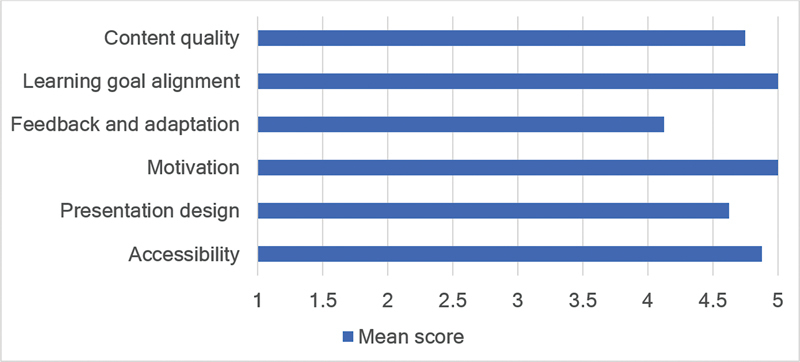
Analysis of the learning object.

The average score of the LO analysis ( Figure 2 ) in all the evaluated items was considered high, between 4.125 and 5.0. It is noteworthy that in two topics of this evaluation, alignment of LOs and motivation, the LO received the maximum score. Accessibility, content quality, and presentation design received mean scores of 4.875, 4.75, and 4.625, respectively. Feedback and Adaptation, despite having a high score of 4.125, received the lowest average among the items studied.

## DISCUSSION

The facial phantom model presents relevant structural characteristics that allow its use in the development of the BoNT application technique in HS and BS. The elastomer tissue that simulates the skin, the anatomical references, and the depth at which BoNT is applied are very similar to the human face. However, despite the analogous structure, there are some limitations, as in any simulation, and the inability to represent the dynamic process of the procedure would be one of them. In this model, as it portrays a younger patient, sagging and facial expression lines commonly seen in patients with BS and HS, more prevalent diseases from the 5th decade of life onwards, 2 8 are not represented. However, there is the possibility of using external anatomical references, well demonstrated in the model, for the development of motor skills. The vascularity normally present in patients, when visualized, can minimally change the direction of the application point. Although this attribute is absent in the simulator, it does not compromise learning the technique, as it does not cause changes in the anatomical reference. Because it is a static object, there are no involuntary reactions or movements at the time of application, which are often seen in procedural practice. The spontaneous contractions evidenced in these pathologies can make the application difficult, however, when a consistent technique is developed, which can be acquired and improved in the learning process, there is less repercussion in the procedure. Despite these limitations, the facial simulator has an affordable price and provides a wide possibility of training and development of competence in the technique of applying BoNT in HS and BS.

The viability of this facial phantom model for the learning process was analyzed by eight physicians with expertise in the use of BoNT. The small number of evaluators is a limitation but reinforces the need to expand teaching in this therapeutic modality. After presenting the simulator model to the selected physicians, they performed a practical test to assess whether this LO could be used in teaching the application technique. Subsequently, these professionals used an adapted instrument for evaluation – LORI. There was great difficulty in obtaining a structured instrument to be used in this assessment, that is, by subjects who already had expertise in the procedure and would analyze the LO. LORI was originally developed for research and evaluation network in LO E-Learning[6]. Although the LO primarily scored by LORI is virtual or digital, conceptually any digital or non-digital entities that can be reused or referenced for learning, education, and training are considered learning objects.

In the LORI analysis, the topics Alignment of LOs and Motivation received the maximum score. Within the context of health education, extrinsic motivation is of great importance for the learning process, and the fact that all professionals with expertise grant the highest score corroborates the LO as valid for the development of this manual skill. Accessibility received an average score of 4.875, which demonstrates the high potential of the LO, due to its portability, weight, and accommodation in different circumstances, to be offered to all medical professionals interested in developing and improving this competence. The Feedback and Adaptation item, despite a high average score of 4.125, received the lowest score among the evaluated aspects. The inference regarding this is the fact that the LO itself does not generate instructions since it is a static object. Content Quality and Presentation Design received average scores of 4.750 and 4.625, respectively. This demonstrates that the LO has a high degree of precision in its form or idea in simulating a human face, the possibility of reusing the product, and providing information for adequate learning.

The Brazilian National Commission for Medical Residency (CNRM) published CNRM Resolution No. 13 on July 6, 2021, which approves the competency matrix of the Medical Residency Programs in Neurology in Brazil. 9 One of the specific objectives outlined in the resolution is the mastery of the BoNT application technique in neurological disorders. However, there is currently no information available in the literature regarding how this technique is taught in medical residencies in neurology, either in Brazil or abroad, especially in developing countries.

The development of the facial simulator phantom model may provide an adequate and broader learning experience, which would lead to less reliance on patients with HS and BS for the development of competence in the BoNT application technique in medical residency in neurology. In addition, the model is cheap, and easy to manufacture and transport, allowing it to be applied in different medical training scenarios, such as neurology, dermatology, plastic surgery, or dentistry. 10

Considering that BoNT is an expensive product with potential adverse reactions in case of misuse, the model guarantees an economic and safe learning experience for health professionals and future patients. However, the model needs to be better tested with a larger number of professionals and compared to other learning strategies for BoNT injections in different residency programs. There is also the possibility of improving the model with anatomical references to the muscles and main vessels of the face, even using resources of augmented reality previously proven successful in vivo applications. 11 A potential limitation of the static prototype is its inability to reproduce some difficulties related to in vivo application, such as moving the face during application, and, obviously, the medical-patient interaction. Additionally, the assessment of the result of the application of botulinum toxin can only be validated through the patient.

In conclusion, the facial phantom model shows promise as a valuable tool for teaching and developing competence in the technique of applying BoNT in HS and BS. While the model has certain limitations, such as the inability to represent dynamic processes and the absence of age-related structural changes, it offers affordable pricing, anatomical references, and a static learning environment. The model was positively evaluated by a group of physicians with expertise in BoNT use, highlighting its potential for teaching and learning.
