# Supplementary material for: Facial phantom model: a low-cost and safe tool for teaching botulinum toxin application in neurology residencies
Source: Arq Neuropsiquiatr. 2024 Jan 29;82(1):s00441779037. doi: 10.1055/s-0044-1779037 (PMC10824594; doi:10.1055/s-0044-1779037)
Supplement: Supplementary file 1 — Supplementary Material [file 10-1055-s-0044-1779037-s230163.pdf]

Supplementary Material

Learning Object Review Instrument - LORI 2.0 adapted

Scoring Sheet

Learning Object ________________________ Reviewer _____________________

General Remarks

Low → **High**

**1. Content Quality:** Accuracy, balanced presentation of ideas, appropriate level of detail, and reuse in various contexts	1	2	3	4	5	NA
**2. Learning Goal Alignment:** Alignment among learning goals, activities, assessments, and learner characteristics.	1	2	3	4	5	NA
**3. Feedback and Adaptation:** Adaptive content or feedback driven by differential learner input or learner modeling	1	2	3	4	5	NA
**4. Motivation:** Ability to motivate and interest an identified population of learners	1	2	3	4	5	NA
**5. Presentation Design:** Design of visual information for enhanced learning and efficient mental processing	1	2	3	4	5	NA
**6. Interaction usability**: Ease of navigation, predictability of the user interface, and the quality of the interface help features	1	2	3	4	5	NA
**7. Accessibility:** Design of controls and presentation formats to accommodate disabled and mobile learners.	1	2	3	4	5	NA
**8. Standards Compliance:** Adherence to international standards and specifications in terms of observance to techncal platforms normally used	1	2	3	4	5	NA

Chart – Additional product information

	Weight	Measures	Costs
Facial Simulator	742,0 g	Height – 21 cm<br>Max Width – 16 cm	R$ 600,00
Acrylic platform	393,0 g	Height (seating position / supine position) - 22/17 cm<br>Width – 17 cm<br>Length – 26 cm	R$ 130,00
Total	1,135 Kg	———————————————————	R$ 730,00
